# Supplementary material for: Seed Germination in Cistus ladanifer: Heat Shock, Physical Dormancy, Soil Temperatures and Significance to Natural Regeneration
Source: Plants (Basel). 2019 Mar 12;8(3):63. doi: 10.3390/plants8030063 (PMC6473532; doi:10.3390/plants8030063)
Supplement: Supplementary file 1 [file plants-08-00063-s001.zip › Table S3.pdf]

Table S3. Weight of seeds of *Cistus ladanifer* not treated with heat before imbibition started (W_s), of non-germinated seeds after no more germination was recorded (W_f) and gain/loss of weight weighed by W_s (Δ).

Replicate and seed number	W_s (mg)	W_f (mg)	Δ (%)	Replicate and seed number	W_s (mg)	W_f (mg)	Δ (%)	Replicate and seed number	W_s (mg)	W_f (mg)	Δ (%)	Replicate and seed number	W_s (mg)	W_f (mg)	Δ (%)
1.1	0.305	0.295	-3.279	2.1	0.264	0.276	4.545	3.1	0.293	a	–	4.1	0.250	a	–
1.2	0.274	0.291	6.204	2.2	0.308	a	–	3.2	0.249	0.252	1.205	4.2	0.337	a	–
1.3	0.258	0.261	1.163	2.3	0.220	0.225	2.273	3.3	0.275	0.270	-1.818	4.3	0.336	0.361	7.440
1.4	0.304	a	–	2.4	0.320	0.335	4.688	3.4	0.293	0.297	1.365	4.4	0.270	0.277	2.593
1.5	0.278	0.305	9.712	2.5	0.268	0.281	4.851	3.5	0.339	a	–	4.5	0.313	a	–
1.6	0.236	0.265	12.288	2.6	0.278	a	–	3.6	0.293	0.331	12.969	4.6	0.281	0.298	6.050
1.7	0.251	b	–	2.7	0.264	0.272	3.030	3.7	0.245	0.255	4.082	4.7	0.284	0.319	12.324
1.8	0.290	0.296	2.069	2.8	0.305	a	–	3.8	0.327	a	–	4.8	0.335	a	–
1.9	0.285	0.282	-1.053	2.9	0.274	0.283	3.285	3.9	0.222	0.244	9.910	4.9	0.265	0.268	1.132
1.10	0.329	0.327	-0.608	2.10	0.263	0.265	0.760	3.10	0.318	a	–	4.10	0.292	0.305	4.452
1.11	0.284	0.305	7.394	2.11	0.247	0.253	2.429	3.11	0.079	c	–	4.11	0.297	0.310	4.377
1.12	0.235	0.239	1.702	2.12	0.264	0.263	-0.379	3.12	0.246	a	–	4.12	0.275	a	–
1.13	0.311	0.312	0.322	2.13	0.296	a	–	3.13	0.278	0.292	5.036	4.13	0.289	0.303	4.844
1.14	0.242	0.250	3.306	2.14	0.276	a	–	3.14	0.286	a	–	4.14	0.207	a	–
1.15	0.325	a	–	2.15	0.082	c	–	3.15	0.246	0.265	7.724	4.15	0.240	0.230	-4.167
1.16	0.320	a	–	2.16	0.274	0.265	-3.285	3.16	0.332	0.348	4.819	4.16	0.311	0.331	6.431
1.17	0.256	0.258	0.781	2.17	0.258	0.282	9.302	3.17	0.331	a	–	4.17	0.247	0.260	5.263
1.18	0.325	a	–	2.18	0.279	0.294	5.376	3.18	0.261	a	–	4.18	0.271	0.293	8.118
1.19	0.253	a	–	2.19	0.265	0.278	4.906	3.19	0.070	c	–	4.19	0.292	0.306	4.795
1.20	0.341	0.346	1.466	2.20	0.347	a	–	3.20	0.281	0.297	5.694	4.20	0.270	0.262	-2.963
1.21	0.292	0.282	-3.425	2.21	0.273	0.283	3.663	3.21	0.291	0.292	0.344	4.21	0.221	a	–
1.22	0.275	0.287	4.364	2.22	0.184	a	–	3.22	0.304	a	–	4.22	0.268	0.284	5.970
1.23	0.220	0.218	-0.909	2.23	0.293	0.288	-1.706	3.23	0.318	0.326	2.516	4.23	0.296	0.305	3.041
1.24	0.306	a	–	2.24	0.311	a	–	3.24	0.304	0.309	1.645	4.24	0.255	0.263	3.137
1.25	0.283	a	–	2.25	0.278	0.292	5.036	3.25	0.305	a	–	4.25	0.312	0.328	5.128

[a] Germinated seed; [b] Lost seed; [c] Empty seed.
