# Supplementary material for: Seed Germination in Cistus ladanifer: Heat Shock, Physical Dormancy, Soil Temperatures and Significance to Natural Regeneration
Source: Plants (Basel). 2019 Mar 12;8(3):63. doi: 10.3390/plants8030063 (PMC6473532; doi:10.3390/plants8030063)
Supplement: Supplementary file 1 [file plants-08-00063-s001.zip › Table S1.pdf]

Table S1. Mean ± SE of final germination of *Cistus ladanifer* seeds incubated under constant and alternate temperatures (16/8 h cycles) and photoperiod (8 h) simultaneous with the highest temperature.

Temperature of incubation (℃)	Photoperiod (h)	Final germination (%)
10	8	30.3±2.1
15	8	37.9±7.6
20	8	27.0±4.1
25	8	27.1±3.6
30	8	21.6±5.0
10/20	8	29.8±2.8
15/25	8	35.7±3.3
20/30	8	30.7±3.5
20	8	27.7±3.4
20	0	28.4±1.9

All samples with n =4.
