# Supplementary material for: Seed Germination in Cistus ladanifer: Heat Shock, Physical Dormancy, Soil Temperatures and Significance to Natural Regeneration
Source: Plants (Basel). 2019 Mar 12;8(3):63. doi: 10.3390/plants8030063 (PMC6473532; doi:10.3390/plants8030063)
Supplement: Supplementary file 1 [file plants-08-00063-s001.zip › Table S2.pdf]

Table S2. Mean ± SE (sample size *n* inside parentheses) of final germination, and of estimated or derived parameters of Weibull equations, lag, rate, duration and shape of germination of *Cistus ladanifer* seeds incubated under constant temperature (20 °C) and photoperiod (8 h) after heat treatments (15 min.).

Temperature of heat treatment (°C)	Final germination (%)	Lag of germination (day)	Rate of germination (day)	Duration of germination (day)	Shape of germination
10	31.7±5.0 (4)	5.0±0.03 (3)	5.8±0.41 (3)	16.8±5.2 (3)	2.9±0.8 (3)
20	27.3±2.5 (8)	4.7±0.99 (5)	4.8±0.55 (5)	15.3±3.3 (5)	3.5±1.2 (5)
30	32.6±1.2 (4)	4.0±0.99 (3)	5.0±0.01 (3)	8.1±0.7 (3)	5.4±0.8 (3)
40	41.8±4.4 (8)	2.8±0.55 (4)	6.3±0.76 (4)	14.2±2.8 (4)	3.6±0.8 (4)
50	34.5±4.5 (4)	3.9±0.07 (2)	4.9±0.07 (2)	10.8±0.5 (2)	3.1±0.1 (2)
60	46.3±8.4 (8)	4.0±0.53 (6)	6.0±0.49 (6)	14.4±2.2 (6)	4.2±1.3 (6)
70	48.4±6.9 (8)	3.1±0.59 (7)	7.9±0.98 (7)	16.9±2.4 (7)	3.5±0.4 (7)
80	89.0±5.5 (4)	2.3±0.20 (4)	10.8±1.76 (4)	21.0±5.3 (4)	4.2±0.7 (4)
90	100.0±0 (4)	2.0±0.03 (4)	7.0±0.55 (4)	9.6±0.2 (4)	8.4±1.7 (4)
100	85.8±7.5 (4)	6.5±0.87 (3)	6.4±1.93 (3)	18.9±2.8 (3)	2.2±0.4 (3)
110	50.9±9.0 (8)	5.5±0.89 (7)	6.1±0.79 (7)	12.9±1.8 (7)	4.1±0.8 (7)
120	26.3±9.8 (8)	9.2±0.91 (3)	4.8±1.95 (3)	12.4±2.9 (3)	2.8±1.1 (3)
130	15.5±10.2 (8)	9.0±1.02 (2)	5.0±1.00 (2)	13.1±0.9 (2)	3.5±0.9 (2)
140	0±0 (4)	–	–	–	–
150	0±0 (4)	–	–	–	–

Differences in sample sizes between final germination and Weibull parameters result from replicates where no germination occurred or from replicates where fitting Weibull equations was not possible.
